# Diallyl trisulfide induces pro-apoptotic autophagy via the AMPK/SIRT1 signalling pathway in human hepatocellular carcinoma HepG2 cell line

**DOI:** 10.29219/fnr.v66.8981

**Published:** 2023-05-19

**Authors:** Shuoshuo Sun, Xiyu Liu, Xiao Wei, Shaohong Zhang, Weimin Wang

**Affiliations:** 1 Affiliated Hospital of Integrated Traditional Chinese and Western Medicine, Nanjing University of Chinese Medicine, Nanjing, China; 2 The Affiliated Huaian NO. 1 People’s Hospital, Nanjing Medical University, Huaian, China

**Keywords:** HepG2 cells, diallyl trisulfide, allicin, apoptosis, autophagy

## Abstract

**Background:**

Liver cancer is associated with a high mortality rate worldwide. Hepatocellular carcinoma (HCC) constitutes a large proportion of primary liver cancers, and most of its alterations currently remain untreatable. Diallyl trisulfide (DATS), the main chemical constituent of allicin, affects tumour development by regulating cell apoptosis. Allicin-induced autophagy could contribute to apoptosis in HepG2 cells. We rigorously examined the autophagy-related mechanism of allicin-induced apoptosis in HepG2 cells. We treated HepG2 cells with DATS to explore the effect of DATS on pro-apoptotic autophagy in HepG2 cell lines and examine its specific molecular mechanism.

**Methods:**

HepG2 cells were treated with various concentrations of DATS for 24 and 48 h. Subsequently, cell viability was measured using the cell counting kit-8 (CCK-8) assay and cell clone formation assay. The HepG2 cell apoptosis was measured using Hoechst 33258 staining and western blotting. Autophagy and the AMP-activated protein kinase (AMPK)/NAD-dependent deacetylase sirtuin-1 (SIRT1) signalling pathway were detected using western blotting.

**Results:**

Our results indicated that DATS inhibited HepG2 cell growth. Moreover, the ability of DATS to promote apoptosis in HepG2 cells increased with increasing concentration. We verified the phenomenon of DATS-induced autophagy in HepG2 cells and demonstrated that DATS treatment upregulated the protein expression of LC3-II/I. By measuring the expression of potential autophagy stimulators, we documented that DATS could induce pro-apoptotic autophagy by activating the AMPK/SIRT1 signalling pathway.

**Conclusion:**

DATS induced pro-apoptotic autophagy via the AMPK/SIRT1 signalling pathway in the human HCC HepG2 cell line. Our findings further implicate allicin as a potential therapeutic agent against liver tumours in clinical settings, providing a basis for combining allicin with an autophagy agonist for treating liver cancer.

## Popular scientific summary

- DATS induces apoptosis in HepG2 cells
- DATS induces autophagy in HepG2 cells
- DATS activates AMPK/SIRT1 signalling in HepG2 cells

Liver cancer is the seventh most frequent malignancy (1), has a high mortality rate and is the fourth leading cause of cancer-related deaths worldwide (1–3). Hepatocellular carcinoma (HCC) reportedly accounts for 80–90% of primary liver cancer cases (2, 4). Currently, the majority of alterations in HCC remain undruggable (4). Treatment based on multi-targeted kinase inhibitor drugs is the current standard first-line therapy, which affords limited survival benefits for patients (5).

Autophagy, a type II form of programmed cell death, is a self-protective mechanism known to be activated in response to nutrient starvation and other stressful stimuli (6, 7). During this dynamic process, cytoplasmic material undergoes separation into autophagosomes and fuses with lysosomes to form autolysosomes, which induce the degeneration or ageing of proteins and defective organelles to lysosomes for degradation (8–11). Autophagy is closely related to human health and disease (12). One of the best-documented cases is cancer, in which pathogenic cellular remodelling is intricately mediated by autophagy (13, 14). It is worth mentioning that autophagy is both a tumour suppressor and a protective factor for cancer cell survival. Thus, autophagy is a double-edged sword in tumorigenesis (15). Autophagy can promote tumourigenesis at all stages, from proliferation to metastasis and invasion, and can facilitate its improvement by providing resistance to death mechanisms (16).

It is well-established that plants of the Allium genus, such as garlic and onions, possess medicinal value (17). However, recent studies have found that garlic does not directly inhibit the growth and spread of cancer cells; however, after cell membrane rupture, alliin can degrade S(+)-allyl-L-cysteine sulfoxide, releasing organic sulphur compounds (18). Allicin is an allyl organic sulphide compound that can be extracted from garlic bulbs. Furthermore, allicin has been shown to significantly impact the treatment of liver cancer-related diseases, indicating its potential as a natural therapeutic substance (19, 20). The main chemical constituent comprising allicin is diallyl trisulfide (DATS), which can modulate disease states such as cancer, metabolic syndrome and infection (21). Accumulating evidence has shown that DATS regulates several cancer-related pathways. For example, DATS affects tumour development by regulating cell apoptosis (17).

Reportedly, allicin-induced autophagy contributes to the apoptosis in HepG2 cells, which involves the p53, mammalian target of rapamycin (mTOR) and AMP-activated protein kinase (AMPK) signalling pathways (7). However, the effect of allicin-induced autophagy on apoptosis and the possible autophagy mechanism of allicin in HCC remain poorly examined; this process may include multiple factors. In the present study, we refined the experimental design and treated HepG2 cells with DATS, aiming to explore the effect of DATS-induced pro-apoptotic autophagy in the HepG2 cell line and determine its specific molecular mechanism.

## Materials and methods

### Reagents and antibodies

High-glucose Dulbecco’s modified Eagle’s medium (DMEM) was purchased from Gibco (USA). Penicillin was purchased from Wanle Pharmaceutical Co. Ltd. (China). Fetal bovine serum (FBS) was purchased from Zhejiang Tianhang Biotechnology Co., Ltd. (China). Dulbecco’s phosphate-buffered saline (D-PBS), phosphate-buffered saline (PBS) and trypsin cell digestion solution were purchased from Gibco (USA). Cell counting kit-8 (CCK-8) was purchased from Dongren Chemical Technology Co., Ltd. (China). DATS was purchased from LKT Laboratories (USA). Dimethyl sulfoxide (DMSO) was purchased from Sigma (USA). Western blot reagents and Hoechst 33258 were purchased from Shanghai Biyuntian Biotechnology Company (China). Bcl-2-associated X protein (Bax), B-cell lymphoma 2 (Bcl-2), MAP1LC3 (LC3), AMPK, phosphorylated AMPK (p-AMPK) and NAD-dependent deacetylase sirtuin-1 (SIRT1) antibodies were purchased from Cell Signalling Technology (USA).

### Sample preparation

The molecular weight and purity of DATS were 178.34 and 99.2%, respectively. The DATS (50 µL) was weighed to calculate the mother liquor concentration, which was 6.27 mol/L. DMSO was used to dilute the DATS concentration to 10, 20, 40, 80, 160 µM in 1.5 mL centrifuge tubes (Corning, USA); the prepared concentrations were stored in a refrigerator (Siemens AG, Germany) at -20°C, protected away from light.

### Cell culture

HepG2 cells were obtained from the Chinese Academy of Sciences (China). Cells were cultured in DMEM complete culture medium, supplemented with 10% FBS, 100 U/mL penicillin and 100 U/mL streptomycin at 37°C in a temperature incubator (Thermol Formal, USA) containing 5% CO_2_.

### Cell proliferation assay

The CCK-8 assay was used to assess the viability of HepG2 cells. Briefly, HepG2 cells were seeded in 96-well plates (Corning, USA) at a density of 1 × 10^5^ cells/well. The cells were treated with different concentrations of DATS (0, 10, 20, 40, 80, 160 µM) for 24 and 48 h at 37°C. Next, 10 μL of the CCK-8 solution was added to cultures and incubated for 2 h at 37°C. Absorbance was measured at 450 nm using a microplate reader (BioTek, USA).

### Cell clone formation assay

HepG2 cells were seeded in a 6-well plate (Corning, USA) at a density of 500 cells per well, with a volume of 50 µL/well. Then, 2 mL of different concentrations (0, 10, 20, 40, 80 and 160 μM) of drug-containing culture medium was added, and the medium was replaced every 3 days. After 3 weeks, the culture medium was removed, and cells were washed with PBS. Subsequently, 4% paraformaldehyde was added to fix the cells for 20 min. After removing the paraformaldehyde, cells were stained with Giemsa staining solution for 30 min, washed with water to remove the staining solution and counted after drying.

### Hoechst 33258 staining

The single-cell suspension (counted and diluted suspension concentration was 1 × 10^5^/mL) was inoculated in a 6-well plate (Corning, USA) and incubated at 37°C for 24 h. After removing the old culture medium from each well, the drug-containing culture medium was added at different concentrations (0, 10, 20, 40, 80 and 160 µM). After incubation at 37°C for 24 h, the drug-containing culture solution was removed. After washing with PBS, 1 mL Hoechst 33258 reagent (10 μg/mL) was added to each well. After incubation for 30 min, the Hoechst reagent was removed. Under 352 nm ultraviolet irradiation, a fluorescence microscope (OLYMPUS, Japan) was used to observe the apoptotic morphology of each well. Five fields were selected for each concentration of culture wells, and the average value of the five fields was used to calculate the cell apoptosis rate (number of apoptotic cells/total number of cells × 100%).

### Western blot analysis

HepG2 cells, treated with different DATS concentrations (0, 40 and 80 µM) for 48 h, were lysed using RIPA buffer (50 mM Tris pH 7.4, 150 mM NaCl, 1% NP-40, 0.5% sodium deoxycholate, 0.1% sodium dodecyl sulphate [SDS], sodium orthovanadate, sodium fluoride, ethylenediaminetetraacetic acid [EDTA] and leupeptin) containing protease inhibitors. The cell lysate was centrifuged at 12,000 *× g* at 4°C for 30 min to pellet the debris, and the protein concentration of the supernatant was estimated using a BCA Kit. Proteins were separated by SDS-polyacrylamide gel electrophoresis (SDS-PAGE) and transferred to a polyvinylidene fluoride (PVDF) membrane. The membranes were incubated overnight at 4°C with primary antibodies specific for Bax, Bcl-2, LC3, AMPK, p-AMPK, SIRT1 and β-actin, followed by incubation with secondary antibodies for 1 h at room temperature. Images were captured using film cassette exposure and chemiluminescence imager exposure (Tanon, China). The relative protein levels were normalised to those of β-actin, used as an internal control.

### Statistical analysis

Data are expressed as the mean ± standard deviation (SD). The Statistical Product and Service Solutions software (SPSS version 22.0) was used for data analyses. Differences amongst groups were determined using a one-way analysis of variance, followed by the post-hoc Dunnett’s *t*-test. Statistical significance was set at *P* < 0.05.

## Results

### DATS inhibits HepG2 cell proliferation

**Fig. 1:**
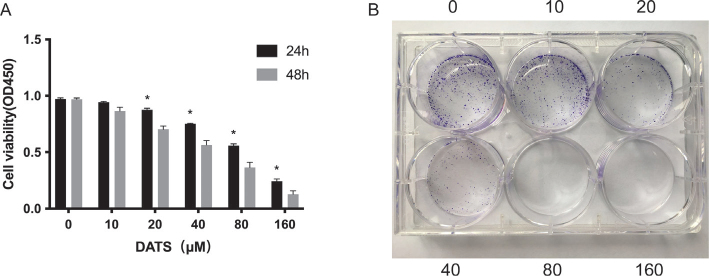
DATS inhibits proliferation in HepG2 cells. (A) The effect of DATS on the cell viability of HepG2. ( *P < 0.05 versus group of different intervention duration with the same treatment concentration). (B) The inhibitory effect of different drug concentrations on the proliferation ability of HepG2.

HepG2 cells were treated with different concentrations of DATS (0, 10, 20, 40, 80, 160 μM) for 24 and 48 h, and cell viability was measured using a CCK-8 assay. The cell viability of each concentration group was time dependent, and cell viability at the same treatment time was dose-dependent (Fig. 1A). Comparing the results of HepG2 cell viability at 24 and 48h, DATS treatment for 48h at 20 μM (*P *<* *0.05), 40 μM (*P *<* *0.05), 80 μM (*P *<* *0.05) and 160* *μM (*P *<* *0.05) reduced cell viability. Thus DATS treatment for 48 h enhanced the inhibition rate. Furthermore, starting from a DATS concentration of 20 μM, the number of viable cells decreased significantly as the DATS concentration increased. Treatment with 80 μM DATS resulted in the absence of viable cells (Fig. 1B).

### DATS induces apoptosis in HepG2 cells

**Fig. 2:**
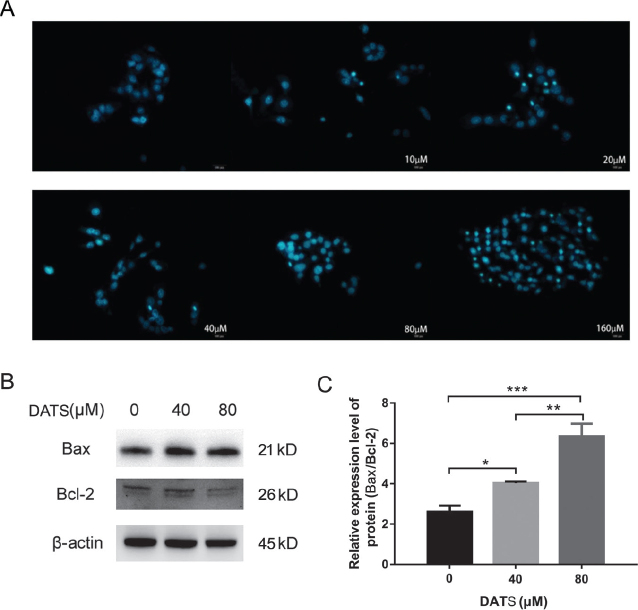
DATS induces apoptosis in HepG2 cells. (A) HepG2 cells apoptosis in each group (view per hole) (Hoechst 33258, 100× magnification). (B and C) Protein expression levels of Bax and Bcl-2. β-Actin was used as an internal control for equal amounts of protein applied. Data are represented as means ± SD; *n* = 3 per group (*P < 0.05, **P < 0.01,***P < 0.001 versus respective control cells).

Compared with the uniformly dyed light-stained particles in the blank group, scattered dark-stained strong blue fluorescent particles were observed in the 10, 20, 40, 80 and 160 µM groups (Fig. 2A). In addition, the 160 μM group exhibited the highest apoptotic rate, whereas the 10 μM group presented the lowest apoptotic rate, showing a concentration-dependent relationship (Table 1).

**Table 1:** Apoptosis rate of HepG2 cells in each group (%)

Group	Apoptosis rate (24 h)
10 µM	3.18 ± 2.25
20 µM	10.28 ± 1.98
40 µM	26.67 ± 2.08
80 µM	40.89 ± 3.35
160 µM	52.48 ± 4.01

The HepG2 cells were treated with various DATS concentrations. Following treatment for 48 h, with increasing DATS concentration, the expression of Bax in liver cancer HepG2 cells showed an upward trend, and the expression of Bcl-2 revealed a downward trend. Furthermore, the Bax/Bcl-2 ratio gradually increased in a concentration-dependent manner (Fig. 2B and C).

### DATS induces autophagy in HepG2 cells

**Fig. 3:**
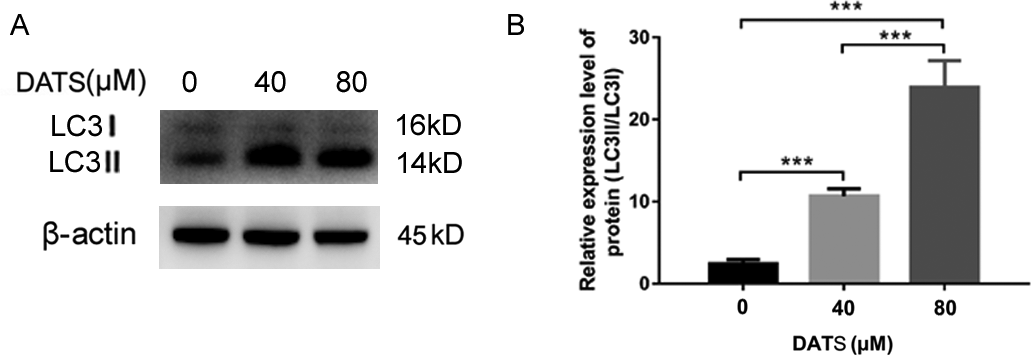
DATS induces autophagy in HepG2 cells. Protein expression level of LC3II-LC3I ratio. β-Actin was used as an internal control for equal amounts of protein applied. Data are represented as means ± SD; *n* = 3 per group (***P < 0.001 versus respective control cells).

To evaluate whether DATS induces autophagy, we treated HepG2 cells with various concentrations of DATS and examined molecular markers of autophagy using western blot analysis. Based on the results, DATS treatment significantly increased the protein expression level of the LC3II-LC3I ratio (Fig. 3).

### DATS activates the AMPK/SIRT1 signalling in HepG2 cells

**Fig. 4:**
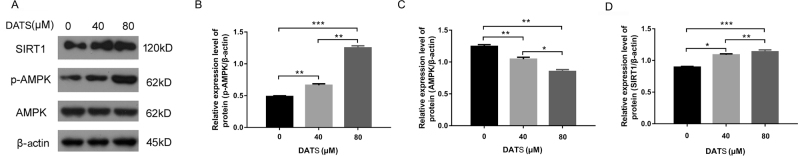
DATS activates AMPK/SIRT1 signalling in HepG2 cells. Protein expression levels of p-AMPK, AMPK and SIRT1. β-Actin was used as an internal control for equal amounts of protein applied. Data are represented as means ± SD; *n* = 3 per group (*P < 0.05, **P < 0.01, ***P < 0.001 versus respective control cells).

Under various stressful conditions, SIRT1 and AMPK are potent stimulators of autophagy in most cells. Western blot analysis was used to determine whether DATS-induced autophagy was mediated by the AMPK/SIRT1 signalling pathway in HepG2 cells. After treatment with various DATS concentrations, the protein expression levels of p-AMPK, AMPK and SIRT1 in HepG2 cells were altered when compared with those in the blank control group. The expression level of p-AMPK protein showed an upward trend and was concentration-dependent (40 μM group *vs.* blank group, *P* < 0.01; 80 μM group *vs.* blank group, *P* < 0.001; 80 μM group *vs.* 40 μM group, *P* < 0.01). There were significant changes in expression levels of the AMPK protein. Compared with the blank group (*P* < 0.01) and the 40 μM group (*P* < 0.05), the AMPK protein expression level of the 80 μM group was significantly reduced; the expression level of the 40 μM group also exhibited a downward trend when compared with the blank group (*P* < 0.01).The expression of SIRT1 showed a notable upward trend, accompanied by a DATS concentration-dependent trend (the SIRT1 expression level in the 80 μM group *vs*. the blank group, *P* < 0.001; the 80 μM group *vs*. the 40 μM group, *P* < 0.01; the 40 μM group *vs*. the blank group, *P* < 0.05). Accordingly, with increasing phosphorylation of AMPK to form p-AMPK, SIRT1 expression also increased (Fig. 4).

## Discussion

The antitumour effect of allicin mainly inhibits cell growth, promotes cell apoptosis and prevents tumour cell metastasis and spread (18). Reportedly, garlic allicin has considerable potential as a novel chemopreventive agent for inhibiting liver cancer (7). Allicin preparations are typically used directly in traditional allicin experiments, and their effective ingredients are poorly defined. The effects and pathways of specific ingredients in allicin preparations on liver cancer HepG2 cells remain unexplored. Therefore, we focused on DATS, the active ingredient in allicin. We examined whether DATS had an inhibitory effect on the growth of HepG2 cells, as well as the mechanism of DATS-induced pro-apoptotic autophagy, and clarified the potential role of the AMPK/SIRT1 signalling pathway.

To clarify whether DATS could inhibit the growth of HepG2 cells, we treated HepG2 cells with different concentrations of DATS. Subsequently, we demonstrated that DATS suppressed liver cancer cell growth using the CCK-8 assay, cell clone formation assay. In addition, we examined the inhibitory effect of DATS on HepG2 cells. All DATS-treated groups exhibited apoptosis of HepG2 cells. This suggested that DATS promoted HepG2 cell apoptosis, and the pro-apoptotic ability increased with increasing concentrations. To determine whether DATS-induced cell death is associated with apoptosis and autophagy, we selected the appropriate DATS concentration and time point and extracted HepG2 cell protein under corresponding conditions to perform a western blot assay. We analysed the associated markers and verified the effects of the AMPK/SIRT1 pathway.

It has been reported that DATS can regulate cell apoptosis and play a role in tumours (17). Herein, the CCK-8 assay confirmed that DATS had an inhibitory effect on human liver cancer HepG2 cells. After determining the dosing time and optimal concentration, DATS concentrations were selected as 0 (blank control group), 40 and 80 μM. After 48 h of treatment with HepG2 cells, the protein was extracted, and the expression levels of Bax and Bcl-2 were measured by western blotting. Bax and Bcl-2 are important apoptotic components. Comparing and analysing grey values, we observed that the expression level of Bax was increased, whereas that of Bcl-2 showed a downward trend. Accordingly, DATS may inhibit tumour cell growth by increasing the expression of Bax in HepG2 cells whilst inhibiting the Bcl-2 expression, thereby inducing apoptosis and killing tumour cells. Combined with the aforementioned evidence regarding allicin, the current study revealed that DATS could inhibit growth and promote apoptosis in the human HCC HepG2 cell line.

Autophagy is an intracellular recycling process that maintains basal levels of metabolites and biosynthetic intermediates under starvation or other forms of stress; therefore, it is an important mechanism for metabolic adaptation in cancer cells (8). LC3 is a key molecular marker protein for autophagy. These findings indicate that DATS could promote autophagy in HepG2 cells. In the present study, DATS treatment upregulated the protein expression of LC3II/LC3I. Allicin was recently identified as an autophagy inducer in HepG2 human liver cancer cells (7). Allicin can increase the protein level of LC3II in HepG2 cells. Treatment of HepG2 human liver cancer cells with allicin could induce LC3II-FITC punctate and colocalization with mitochondria. In addition, Atg7, TSC2 and Beclin-1 have shown similar results to those of pharmacological inhibition, and allicin-induced colocalization of LC3-II-FITC punctate and mitochondria could be suppressed by 3-MA (autophagy inhibitor) pretreatment, which confirmed the induction of autophagy by allicin in HepG2 human liver cancer cells (7). Other detailed studies have examined autophagy. Accordingly, we refined the research objective to further verify the phenomenon of DATS-induced autophagy in HepG2 cells. It must be emphasised that our focus was to explore the relevant mechanisms.

AMPK is a major energy sensor that controls cellular metabolism and energy homeostasis (7). Given its key role in controlling energy homeostasis, AMPK has attracted widespread attention as a potential therapeutic target for metabolic diseases (22). AMPK regulates energy levels, enhances metabolic checkpoints and inhibits cell growth, possibly by inhibiting tumour metabolism. Several studies have demonstrated the tumour suppressor function of AMPK in lung, colorectal and liver cancers (23). AMPK can inhibit HCC by inducing cell senescence and autophagy (24). AMPK promotes autophagy via phosphorylation (25). In HepG2 cells, allicin can significantly increase AMPK phosphorylation in 1,3-dichloro-2-propanol-induced lipid metabolism disorder (26).

Histone deacetylases (HDACs) play a key role in regulating various events in tumour cells, and studies have shown that HDACs are abnormally expressed during tumorigenesis (27). In HDACs, the sirtuin protein family (SIRT1-SIRT7) plays a unique and important role. SIRT1, a homologue of silent information regulator 2, is an NAD^+^-dependent deacetylase expressed in all tissues (28). SIRT1 can alter its metabolic programme via deacetylation in response to diverse physiological stresses and is considered a key regulator of various biological processes (27–29). SIRT1 is associated with multiple diseases, including cancer, vascular diseases and neurodegenerative disorders (12). In addition, SIRT1 plays a role in gene regulation, genome stability maintenance, apoptosis, autophagy, senescence, proliferation, and tumourigenesis (30, 31).

Following phosphorylation, AMPK can activate SIRT1 by altering the ratio of NAD^+^/NADH. In the present study, after 48 h of DATS treatment, we found that with increasing DATS concentration, the level of p-AMPK increased significantly, as determined by western blotting. Accordingly, AMPK undergoes activation and phosphorylation. Simultaneously, the level of SIRT1 also showed an upward trend, consistent with the expression of p-AMPK. Based on this experiment, the pro-apoptotic autophagy effect mediated by DATS on HepG2 cells may be related to the AMPK/SIRT1 signalling pathway.

Reportedly, allicin induces p53-mediated autophagic cell death in liver cancer cells by activating the p-AMPK and TSC2 signalling pathways and inhibiting the mTOR and cytoplasmic p53 signalling pathways (7). We speculate that SIRT1 is also directly involved in the process of autophagy flow formation; therefore, we conducted an in-depth assessment of signalling pathways to explore the role of AMPK-SIRT1 in HepG2 autophagy. SIRT1 can be modified by deacetylation to regulate p53 activity. Therefore, the observed phenomenon could explain how DATS regulates the activity of p53 via AMPK.

Previous studies have reported the effects of allicin intervention by adding AMPK inhibitors to HepG2 cells. The expression of AMPK and p-AMPK was detected by pre-incubating HepG2 cells for 1 h in the absence or presence of an AMPK inhibitor (Compound C). Pretreatment with an AMPK inhibitor could inhibit the restorative effect of allicin on p-AMPK expression in HepG2 cells (26). This result supports the hypothesis that AMPK inhibitors, to a certain extent, can affect autophagy following DATS treatment. However, given that no AMPK/SIRT1 inhibitor was added to confirm the role of AMPK/SIRT1 in DATS-mediated inhibition of HepG2 cell growth in our current experimental study, we cannot exclude the possibility that AMPK phosphorylation can increase SIRT1 and jointly inhibits the growth of HepG2 cells. Further experiments are warranted to confirm this hypothesis.

In summary, our study provides evidence that DATS induces pro-apoptotic autophagy by activating the AMPK/SIRT1 signalling pathway. These findings support the notion that allicin may represent a potential therapeutic candidate for treating HCC. The possibility of employing allicin as an anti-liver tumour agent in clinical settings has greatly increased. Moreover, our study may provide a new strategy for treating liver cancer, using combined therapy with allicin and autophagy agonists.

## Conflict of interest and funding

The authors have declared no conflict of interest. The authors have not received any benefits from industry or elsewhere to conduct this study.

## References

[1] Bray F, Ferlay J, Soerjomataram I, Siegel RL, Torre LA, Jemal A. Global cancer statistics 2018: GLOBOCAN estimates of incidence and mortality worldwide for 36 cancers in 185 countries. CA Cancer J Clin 2018; 68(6): 394–424. doi: 10.3322/caac.21492 30207593

[2] Li X, Ramadori P, Pfister D, Seehawer M, Zender L, Heikenwalder M. The immunological and metabolic landscape in primary and metastatic liver cancer. Nat Rev Cancer 2021; 21(9): 541–57. doi: 10.1038/s41568-021-00383-9 34326518

[3] Llovet JM, Kelley RK, Villanueva A, Singal AG, Pikarsky E, Roayaie S, et al. Hepatocellular carcinoma. Nat Rev Dis Primers 2021; 7(1): 6. doi: 10.1038/s41572-020-00240-3 33479224 PMC13537433

[4] Wang C, Cao Y, Yang C, Bernards R, Qin W. Exploring liver cancer biology through functional genetic screens. Nat Rev Gastroenterol Hepatol 2021; 18(10): 690–704. doi: 10.1038/s41575-021-00465-x 34163045

[5] Kudo M, Finn RS, Qin S, Han KH, Ikeda K, Piscaglia F, et al. Lenvatinib versus sorafenib in first-line treatment of patients with unresectable hepatocellular carcinoma: a randomised phase 3 non-inferiority trial. Lancet 2018; 391(10126): 1163–73. doi: 10.1016/S0140-6736(18)30207-1 29433850

[6] Chen N, Karantza-Wadsworth V. Role and regulation of autophagy in cancer. Biochim Biophys Acta 2009; 1793(9): 1516–23. doi: 10.1016/j.bbamcr.2008.12.013 19167434 PMC3155287

[7] Chu YL, Ho CT, Chung JG, Rajasekaran R, Sheen LY. Allicin induces p53-mediated autophagy in Hep G2 human liver cancer cells. J Agric Food Chem 2012; 60(34): 8363–71. doi: 10.1021/jf301298y 22860996

[8] Yu L, Chen Y, Tooze SA. Autophagy pathway: cellular and molecular mechanisms. Autophagy 2018; 14(2): 207–15. doi: 10.1080/15548627.2017.1378838 28933638 PMC5902171

[9] Ba L, Gao J, Chen Y, Qi H, Dong C, Pan H, et al. Allicin attenuates pathological cardiac hypertrophy by inhibiting autophagy via activation of PI3K/Akt/mTOR and MAPK/ERK/mTOR signaling pathways. Phytomedicine 2019; 58: 152765. doi: 10.1016/j.phymed.2018.11.025 31005720

[10] Lai L, Chen J, Wang N, Zhu G, Duan X, Ling F. MiRNA-30e mediated cardioprotection of ACE2 in rats with Doxorubicin-induced heart failure through inhibiting cardiomyocytes autophagy. Life Sci 2017; 169: 69–75. doi: 10.1016/j.lfs.2016.09.006 27633839

[11] Xia P, Liu Y, Cheng Z. Signaling pathways in cardiac myocyte apoptosis. Biomed Res Int 2016; 2016: 9583268. doi: 10.1155/2016/9583268 28101515 PMC5215135

[12] Guo H, Ding H, Tang X, Liang M, Li S, Zhang J, et al. Quercetin induces pro-apoptotic autophagy via SIRT1/AMPK signaling pathway in human lung cancer cell lines A549 and H1299 in vitro. Thorac Cancer 2021; 12(9): 1415–22. doi: 10.1111/1759-7714.13925 33709560 PMC8088950

[13] Gu J, Hu W, Song ZP, Chen YG, Zhang DD, Wang CQ. Rapamycin inhibits cardiac hypertrophy by promoting autophagy via the MEK/ERK/Beclin-1 pathway. Front Physiol 2016; 7: 104. doi: 10.3389/fphys.2016.00104 27047390 PMC4796007

[14] Li L, Xu J, He L, Peng L, Zhong Q, Chen L, et al. The role of autophagy in cardiac hypertrophy. Acta Biochim Biophys Sin (Shanghai) 2016; 48(6): 491–500. doi: 10.1093/abbs/gmw025 27084518 PMC4913516

[15] Chen N, Karantza V. Autophagy as a therapeutic target in cancer. Cancer Biol Ther 2011; 11(2): 157–68. doi: 10.4161/cbt.11.2.14622 21228626 PMC3230307

[16] Kocaturk NM, Akkoc Y, Kig C, Bayraktar O, Gozuacik D, Kutlu O. Autophagy as a molecular target for cancer treatment. Eur J Pharm Sci 2019; 134: 116–37. doi: 10.1016/j.ejps.2019.04.011 30981885

[17] Puccinelli MT, Stan SD. Dietary bioactive diallyl trisulfide in cancer prevention and treatment. Int J Mol Sci 2017; 18(8): 1645. doi: 10.3390/ijms18081645 28788092 PMC5578035

[18] Bocchini P, Andalo C, Pozzi R, Galletti GC, Antonelliet A. Determination of diallyl thiosulfinate (allicin) in garlic (Allium sativum L.) by high-performance liquid chromatography with a post-column photochemical reactor. Analytica Chimica Acta 2001; 441(1): 37–43. doi: 10.1016/S0003-2670(01)01104-7

[19] Li X, Haut RC, Altiero NJ. An analytical model to study blunt impact response of the rabbit PF joint. Journal of biomechanical engineering 1995; 117(4): 485–91. doi: 10.1115/1.2794212 8748533

[20] Ossama M, Hathout RM, Attia DA, Mortada ND. Enhanced allicin cytotoxicity on HEPG-2 cells using glycyrrhetinic acid surface-decorated gelatin nanoparticles. ACS Omega 2019; 4(6): 11293–300. doi: 10.1021/acsomega.9b01580 31460232 PMC6648216

[21] Mikaili P, Maadirad S, Moloudizargari M, Aghajanshakeri S, Sarahroodi S. Therapeutic uses and pharmacological properties of garlic, shallot, and their biologically active compounds. Iran J Basic Med Sci 2013; 16(10): 1031–48. PMID: 24379960 PMC3874089

[22] Cool B, Zinker B, Chiou W, Kifle L, Cao N, Perham M, et al. Identification and characterization of a small molecule AMPK activator that treats key components of type 2 diabetes and the metabolic syndrome. Cell Metab 2006; 3(6): 403–16. doi: 10.1016/j.cmet.2006.05.005 16753576

[23] Umezawa S, Higurashi T, Nakajima A. AMPK: therapeutic target for diabetes and cancer prevention. Curr Pharm Des 2017; 23(25): 3629–44. doi: 10.2174/0929867324666170713150440 28714409

[24] Hu M, Huang H, Zhao R, Li P, Li M, Miao H, et al. AZD8055 induces cell death associated with autophagy and activation of AMPK in hepatocellular carcinoma. Oncol Rep 2014; 31(2): 649–56. doi: 10.3892/or.2013.2890 24297300

[25] Kim J, Kundu M, Viollet B, Guan KL. AMPK and mTOR regulate autophagy through direct phosphorylation of Ulk1. Nat Cell Biol 2011; 13(2): 132–41. doi: 10.1038/ncb2152 21258367 PMC3987946

[26] Lu J, Cheng B, Fang B, Meng Z, Zheng Y, Tian X, et al. Protective effects of allicin on 1,3-DCP-induced lipid metabolism disorder in HepG2 cells. Biomed Pharmacother 2017; 96: 1411–7. doi: 10.1016/j.biopha.2017.10.125 29169723

[27] Dong YJ, Liu N, Xiao Z, Sun T, Wu SH, Sun WX, et al. Renal protective effect of sirtuin 1. J Diabetes Res 2014; 2014: 843786. doi: 10.1155/2014/843786 25386563 PMC4214106

[28] Qiang L, Wang L, Kon N, Zhao W, Lee S, Zhang Y, et al. Brown remodeling of white adipose tissue by SirT1-dependent deacetylation of Pparγ. Cell 2012; 150(3): 620–32. doi: 10.1016/j.cell.2012.06.027 22863012 PMC3413172

[29] Guarente L, Picard F. Calorie restriction--the SIR2 connection. Cell 2005; 120(4): 473–82. doi: 10.1016/j.cell.2005.01.029 15734680

[30] Zhang S, Zhang M, Sun S, Wei X, Chen Y, Zhou P, et al. Moderate calorie restriction ameliorates reproduction via attenuating oxidative stress-induced apoptosis through SIRT1 signaling in obese mice. Ann Transl Med 2021; 9(11): 933. doi: 10.21037/atm-21-2458 34350248 PMC8263864

[31] Alves-Fernandes DK, Jasiulionis MG. The role of SIRT1 on DNA damage response and epigenetic alterations in cancer. Int J Mol Sci 2019; 20(13): 3153. doi: 10.3390/ijms20133153 31261609 PMC6651129

